# Supporting Witnesses and Victims to Invoke Episodic Retrieval Mode: Own-Generated Verbal- and Sketch-Reinstatement-of-Context Retrieval Cues Improves Recall Versus Interviewer-Generated Mental Reinstatement of Context Cues

**DOI:** 10.3390/bs16020245

**Published:** 2026-02-09

**Authors:** Coral J. Dando, Rachael V. Dando, Hannah Richardson, Aurora Osorio Rojas, Donna A. Taylor

**Affiliations:** 1 Department of Psychology, University of Westminster, London W1W 6UW, UK; hannah.richardson1591@gmail.com (H.R.); auroraosorio1998@gmail.com (A.O.R.); d.taylor4@westminster.ac.uk (D.A.T.); 2 Mental Health Treatment Requirement Services, Mersey Care NHS Foundation Trust, Liverpool L1 9AA, UK; rachael.dando@merseycare.nhs.uk

**Keywords:** eyewitness memory, Sketch-Reinstatement-of-Context, self-generated retrieval cues, interviewer-generated retrieval cues, episodic retrieval mode

## Abstract

Background: Criminal justice relies on information from witnesses. Retrieval from episodic memory is cognitively demanding; thus, many interview protocols advocate techniques to support episodic retrieval mode, which is essential for obtaining detailed accounts. Currently, interviewers have two empirically validated techniques for triggering and scaffolding conscious remembering: Mental-Reinstatement-of-Context and Sketch-Reinstatement-of-Context. However, where neither is appropriate, there exist few alternatives. We report a potential future addition to the interviewer toolbox, aimed at reinstating context through self-directed verbal cueing, namely the Verbal-Reinstatement-of-Context. Methods: Using a between-conditions mock witness paradigm, we compared the interviewer-directed Mental-Reinstatement-of-Context technique with self-directed Sketch-Reinstatement-of-Context and Verbal-Reinstatement-of-Context cue techniques. Participants were interviewed 48 h after they had seen a mock robbery. Memory performance was analyzed for correct and erroneous recall, completeness, and accuracy. Results: Participants who self-generated retrieval cues recalled an average of 26% (Sketch-Reinstatement-of-Context) and 11% (Verbal-Reinstatement-of-Context) more correct information and were more complete and more accurate than those in the Mental-Reinstatement-of-Context condition. Improved recall was not accompanied by increased errors. Mean combined errors were an average of 34% and 22% lower (respectively) in the self-generated cue conditions. Conclusions: Consistent with prior research, self-generated retrieval cues were more effective than interviewer-initiated cues. Sketch-Reinstatement-of-Context and Verbal-Reinstatementme-of-Context conferred clear advantages, although Sketch-Reinstatement-of-Context was most efficient overall. For witnesses unable or unwilling to sketch, Verbal-Reinstatement-of-Context may be a viable alternative.

## 1. Introduction

Episodic memory concerns the recollection of personally experienced events, which include salient contextual information specific to individuals, the timing of the event, and the place of acquisition (Tulving, 2002; Tulving & Terrace, 2005). Criminal justice systems (CJS) worldwide rely on episodic accounts from witnesses^1^ who are asked to explain what they have seen and heard in as much detail as possible, usually during a formal interview with a police officer or other investigation professional. In recognition that retrieval from episodic memory is cognitively demanding, best practice witness interview protocols, such as the cognitive interview (Fisher & Geiselman, 1992), advocate the use of mnemonic techniques to support episodic retrieval mode. Episodic retrieval mode is a neurocognitive state when medial temporal lobe and prefrontal cortex networks are active, supporting memory search and self-referential processing (e.g., Danker & Anderson, 2010; Lepage et al., 2000; Rugg & Vilberg, 2013). This state facilitates contextual recall of past events, which is essential for obtaining detailed and accurate witness accounts.

One of the core cognitive interview mnemonics that can support episodic retrieval mode is the mental reinstatement of context technique (MRC). MRC was devised to encourage witnesses to mentally re-create the psychological, physiological, and physical encoding environment towards facilitating the feature overlap between the to-be-remembered event and the retrieval environment (see Schacter & Tulving, 1982; Tulving & Thomson, 1973). Contextual information (i.e., spatial, temporal, context, and cognitive details) encoded alongside the target information is important for triggering and scaffolding conscious remembering of personally experienced events (e.g., Hennings et al., 2019; Polyn et al., 2009, 2005; Smith & Vela, 2001). Indeed, the theoretical and applied psychological literature reveals how supporting the reinstatement of contextual elements at retrieval can significantly improve witness memory (e.g., Choi et al., 2025; Bramao et al., 2017; C. J. Dando et al., 2020, 2023a; M. Mattison et al., 2018; Maras et al., 2020; Murnane et al., 1999; Smith & Vela, 2001; Torres-Morales & Cansino, 2024).

The beneficial effects of witness interview protocols that include the MRC technique versus a control interview that does not include MRC typically include more complete recall and fewer errors (e.g., C. Dando et al., 2009; C. J. Dando et al., 2011, 2020; M. L. Mattison et al., 2015; M. Mattison et al., 2018; Memon et al., 2010; Milne & Bull, 2002). However, MRC is a cognitively demanding technique and as such is inappropriate for some populations, including traumatized individuals, older adults and neurodivergent children and adults, since cognitive demand can outstrip cognitive resources available and MRC can evoke vivid imagery, physiological arousal, and intrusive emotional memories, similar to trauma re-experiencing described in PTSD models (Brewin, 2014; C. J. Dando, 2013; C. J. Dando et al., 2020, 2023a; Ehlers & Clark, 2000; Maras et al., 2020; M. L. Mattison et al., 2015; M. Mattison et al., 2018; Mystkowski et al., 2006). MRC also relies heavily on the generation of visual imagery in response to interviewer provided, requiring witnesses to mentally reconstruct and simulate prior experiences. This process draws on episodic retrieval mechanisms and imagery-based working memory resources, which are finite and capacity-limited. The effortful construction and maintenance of mental images places additional demands on attentional control and executive processes, particularly when multiple cues must be integrated or when recall involves complex or emotionally salient events. As a consequence, the cognitive resources available for accurate retrieval, monitoring, and verbalization of memory content may be reduced, potentially constraining recall completeness or accuracy under conditions of high cognitive load (e.g., Conway & Pleydell-Pearce, 2000; Gomes et al., 2025; Greenberg & Knowlton, 2014; Keogh & Pearson, 2014; Rubin, 2006).

There is increasing evidence that visual imagery is not experienced in a universal or uniform capacity. Rather, people differ in their ability to generate, maintain, and manipulate mental images. For example, individuals with aphantasia report little or no voluntary visual imagery. These individual differences are stable, measurable, and associated with variation in episodic memory retrieval, autobiographical recall, and imagery-dependent cognitive tasks. Consequently, interview techniques that rely heavily on imagery-based reconstruction may confer uneven benefits and may impose disproportionate cognitive demands on individuals with limited imagery capacity (C. J. Dando et al., 2023a; Palombo et al., 2015; Pearson, 2019; Zeman et al., 2020). Further, interviewer-initiated retrieval cues in the MRC technique are unlikely to be universally salient. The effectiveness of retrieval cues depends on the idiosyncratic encoding conditions. Cues selected by an interviewer may therefore fail to align with personally salient perceptual, contextual, or emotional features, rendering them weak or ineffective triggers for retrieval. What constitutes a meaningful cue for one witness may be irrelevant—or even distracting—for another, potentially increasing cognitive load without facilitating recall (e.g., Conway & Pleydell-Pearce, 2000; C. Dando et al., 2009; C. J. Dando, 2013; Tulving & Thomson, 1973). From a practitioner perspective, MRC is also time-consuming and can be challenging to apply in terms of the complexity of the instructions, which can overburden interviewers (Bangs & Smith-Spark, 2020; C. Dando et al., 2008, 2009).

One alternative to MRC that has been found to be effective for supporting episodic retrieval mode is sketching (e.g., C. J. Dando, 2013; C. Dando et al., 2009; C. J. Dando et al., 2011, 2020, 2023a; Eastwood et al., 2018; Luther et al., 2023; M. L. Mattison et al., 2015; M. Mattison et al., 2018). The Sketch-Reinstatement-of-Context technique (Sketch-RC) does not rely on visual imagery and does require interviewers to provide retrieval cues, but instead asks witnesses to draw ‘*whatever reminds you about what happened*’. The process of drawing personally relevant event elements at the outset of an interview appears to encourage a more effortful and elaborative retrieval search than might otherwise occur. By actively constructing a visual representation, witnesses self-generate personally salient contextual cues that reflect their original encoding experience, potentially increasing the encoding–retrieval overlap by reinstating idiosyncratic features of the event, thereby facilitating access to episodic details. Consistent with research, self-generated representations promote deeper processing and richer cue–target associations, resulting in improved memory performance relative to more passive or verbally constrained retrieval approaches (e.g., Fernandes et al., 2018; Tran et al., 2023; Wammes et al., 2018). Indeed, Sketch-RC has been found to improve recall accuracy and the total of correct event information by witnesses with no concomitant increase in errors versus interviews incorporating MRC and control interviews where no retrieval support is offered (C. J. Dando, 2013; C. Dando et al., 2009; C. J. Dando et al., 2020; Matsuo & Miura, 2017; M. L. Mattison et al., 2015; M. Mattison et al., 2018). In the case of the latter two conditions, Sketching has recently been integrated into best practice guidance in England and Wales as a suitable technique for use when interviewing vulnerable witness populations (Ministry of Justice, 2022).

We have previously discussed how, as with MRC, the Sketch-RC is unsuitable for some witness populations. Although Sketch-RC is simple to use, the technique may be impossible for some witnesses with physical or sensory disabilities who may be unable to draw. It has also been our experience that when interviewing some adults and older adults (as research participants and in professional forensic contexts), a very small number have indicated some discomfort at being asked to sketch, citing general concerns/embarrassment associated with drawing/sketching in the presence of others. Despite explaining the cognitive benefits of sketching and the fact that the sketch or drawing itself is less important, alongside encouraging witnesses to draw, social discomfort was clear on occasion. Witness interviews are socially and cognitively demanding, *per se*, and so additional social demands associated with techniques that are designed to assist witnesses will undoubtedly be counterproductive (e.g., C. J. Dando et al., 2023b; May et al., 2025; Sweller et al., 2011; Zloteanu & Porter, 2025). The development and maintenance of rapport may be disrupted, and socially uncomfortable witnesses are less likely to fully engage with the interview process, a corollary being impoverished recall (Collins et al., 2002; Gabbert et al., 2021; Nahouli et al., 2021; Vallano & Compo, 2011). Thus, from a witness compatible interaction perspective, and towards widening access to criminal justice for those who are unable to sketch, developing additional techniques for supporting episodic retrieval mode is vital.

Both MRC and Sketch-RC provide witness-compatible options for interviewers who can choose the most appropriate from a *toolbox* of validated techniques. However, in instances where neither is appropriate (for whatever reason), as far as we are aware, there exist few alternatives. Yet, the extant theoretical and applied psychological literature is consistent in reporting that appropriate external retrieval support improves the accuracy and completeness of witness accounts. Impoverished, erroneous, and episodic accounts reduce access to justice for witnesses and contribute to failed investigations and even wrongful convictions (e.g., Geven et al., 2025; Loftus & Greenspan, 2017; Wells & Olson, 2003; Vrij et al., 2014).

Here, we report research investigating a potential future addition to the interviewer toolbox, aimed at reinstating context through self-directed verbal cueing, henceforth referred to as Verbal-RC. Recollection-driven recall involves reinstatement of encoding-related activity at the time of retrieval, which can be supported externally by the MRC technique using mental imaging and by the Sketch-RC method via drawing or sketching. The Verbal-RC technique differs in that, although it asks witnesses to think back to the to-be-remembered event, reinstatement of encoding-related activity is triggered by being asked to say out loud the first word that comes to mind (see materials and methods for a full description). These words (in some cases, witnesses said two words, for example, car accident) are noted by the interviewer. This instruction is then repeated an additional four times. Each additional word/words are noted by the interviewer in order. As with MRC and Sketch-RC, Verbal-RC is used to trigger episodic retrieval mode prior to an initial free recall and subsequent probing questions.

The Task Support Hypothesis (e.g., Bowler et al., 2015) contends that performance can be improved when retrieval support is uncomplicated and where sufficient time is allowed to process cognitive tasks (e.g., Ballesteros et al., 2013; Colcombe & Kramer, 2003). Verbal-RC naturally allows witness compatible adjustments in line with the Task Support Hypothesis. The instructions are few and simple, allowing witnesses to dictate the pace of recall, thereby reducing situational demands. Research also suggests self-generated cues are typically more effective for cueing the retrieval of to-be-remembered events since they include idiosyncratic aspects of the encoding context, which are personal and individually salient (e.g., C. J. Dando et al., 2020; Wheeler & Gabbert, 2017). Thus, both Sketch-RC and Verbal-RC confer similar advantages since both allow self-generated cues and support witness-compatible adjustments in timing and pace. However, drawing engages both visual-spatial and verbal memory systems (e.g., Zimmer et al., 2007; Zimmer, 2008), which may trigger richer contextual reinstatement through multimodal retrieval processes. Therefore, theoretically and empirically, it is reasonable to expect Sketch-RC to outperform Verbal-RC, which relies purely on verbal cueing for episodic memory retrieval. Further, given current understanding of the cognitive demand associated with the MRC technique, which relies on other-generated cues, we expect both the Sketch-RC and Verbal-RC to improve recall performance (remembering more event information without a concomitant increase in erroneous recall) versus the MRC since both rely on self-generated retrieval cues, which are immediately externalized either verbally or by sketching.

A series of ANOVAs was conducted, followed by planned comparisons for global (from the start to the end of the interview) memory performance measures (correct, errors, confabulations, and percentage accuracy). Memory performance measures include the amount of unique correct information items recalled, the number of errors, completeness, and percentage accuracy. We formulated the following hypotheses with a focus on global performance. First, (H^1^) both self-generated retrieval cue conditions (Sketch-RC and Verbal-RC) would improve mock witness recall of the target event versus the other-generated cue condition (MRC). Second, (H^2^) there would be a significant difference in memory performance across the two self-generated cue conditions (Sketch-RC and Verbal-RC), whereby Sketch-RC would improve memory performance versus Verbal-RC. Additional analysis of interview phase performance across the three retrieval conditions was conducted, followed by post hoc tests to understand the impact of the experimental manipulations on each of the retrieval phases of an investigative interview in isolation, namely the free recall and probing questioning phases.

## 2. Materials and Methods

### 2.1. Design

A between-subjects experimental design was employed with one independent variable (external retrieval support) on three levels: (i) Mental-Reinstatement-of-Context (MRC), (ii) Sketch-Reinstatement-of-Context (Sketch-RC), and (iii) Verbal-Reinstatement-of-Context (Verbal-RC). The dependent variables were recall performance measured globally (across the entire interview from the start of the first retrieval phase to the end of the interview) and as a function of recall phase (free recall and probed recall) for correct, erroneous, and confabulated information items verbalized percentage accuracy and completeness (see procedure). Post-interview feedback data were collected from participants via a questionnaire completed within 30 min following participation.

This research was approved by the University of Westminster Ethics Committee (ETH20210827) and adhered to the Health and Care Practitioner Council and British Psychological Society’s code of conduct. Data were handled in line with regulatory requirements. Informed consent was acquired prior to participation, and a debrief of the study manipulation was provided at the end of the study.

### 2.2. Participants

A total of 149 adults from the general population took part in the research as mock witnesses, 63 males and 86 females, with a mean age of 30.24 years (SD = 6.05 years), ranging from 20 to 41 years. Participants were recruited via social media, snowballing, and convenience sampling, and were randomly assigned to one of the three retrieval conditions (i) Mental-Reinstatement-of-Context (MRC), (ii) Sketch-Reinstatement-of-Context (Sketch-RC), and (iii) Verbal-Reinstatement-of-Context (Verbal-RC). There was a non-significant mean age difference across conditions *F*(2, 149) = 0.120, *p* = 0.887 (*M*_Sketch-RC_ = 30.23, SD = 5.71; *M*_MRC_ = 29.93 SD = 6.89; *M*_Verbal-RC_ = 30.54 SD = 5.61). A total of 48 participants (32.2%) were allocated to the MRC condition, 51 (34.2%) to the Sketch-RC, and 50 (33.6%) to the Verbal-RC condition. A priori power analysis for three independent groups ANOVA fixed effects indicated that a sample size of 155 participants was required to achieve a power of 0.80, with an alpha level of 0.05, and a medium effect size (Faul et al., 2007).

### 2.3. Mock Crime Stimulus Video

A pre-recorded video lasting 56 s was viewed by participants individually. The film shows a shop robbery involving two people who are initially seen walking down the road past a parade of shops and into one of the shops. Approximately 20 s later, the same two people run out of the shop, chased by a man (the shop owner) who is shouting at them. The video ends.

### 2.4. Interview Protocol

Irrespective of condition, all interviews conducted for this research were based on the current guidance for gathering witness information in ‘volume crime’ applied forensic contexts in the UK, and across many European jurisdictions. Volume crime refers to frequently occurring offenses in the UK that significantly impact communities, such as burglary, theft, shoplifting, robbery, vehicle crime, and assault. Each interview comprised two recall phases, namely a free recall and a probing questioning phase. Probing questions were asked by the interviewer during this second recall phase, only about topics recalled in the initial free recall phase. All interviews were similarly structured, comprising the following phases: (i) greet, explain, & rapport, (ii) free recall (recall 1), (iii) probing questions (recall 2), and (iv) closure. Interviews comprised the same number of retrievals in the same order but differed at the commencement of the free recall phase (only) according to condition, since it was at the start of this first recall phase that experimental manipulations took place.

Six female researchers (naïve to the research hypotheses) with a mean age of 33 years (ranging from 29 to 40 years) conducted all interviews. Each interviewer conducted between 14 and 36 interviews across all three conditions, following the condition-appropriate protocols (verbatim) as follows (detailed interview protocols are available from the first author).

i. In the greet, explain, & rapport phase (common to all interviews), interviewers greeted the participant, introduced themselves, and explained what the interview would entail. Each participant was given an opportunity to ask any questions, and permission was sought for each interview to be digitally recorded. Throughout, the interviewer interacted with the participant, contributing as an interested party, using open-ended invitations and associated verbal (e.g., adherence to the interview protocol, and referring to the participant by name, thanking the participant for explaining what they remember, etc.) and physical behaviors (nodding, smiling, looking at the participant when they are talking, and generally being attentive) to exchange information and to demonstrate an understanding of the situation from the participant’s point of view (e.g., C. J. Dando et al., 2023b; Nahouli et al., 2021; Vallano & Compo, 2011).
ii. The free recall phase (common to all interviews) invites participants to ‘explain’ everything they can recall about the experienced event using an open-ended invitation. This differed across conditions as follows:
  - In the Sketch-RC condition, participants were first asked to draw the to-be-remembered event in as much detail as possible (see C. J. Dando et al., 2020; C. Dando et al., 2009; C. J. Dando, 2013). Participants were asked to draw anything that reminded them of what happened. Participants were given unlimited time to draw. Once participants had finished, the interviewer then asked to ‘tell me absolutely everything you can remember about what happened’, but (i) I only want you to tell me what you actually remember, please don’t guess, (ii) if you can’t remember just say so, (iii) tell me absolutely everything you can, even if you can only remember partial details, or apparently insignificant information, and (iv) tell me if you do not understand what I am asking or to repeat the question (from here on these instructions are referred to as the retrieval instructions). Participants were invited to refer to their sketches if they wished (see C. J. Dando, 2013; C. J. Dando et al., 2023a; M. Mattison et al., 2018).
  - In the MRC condition, the interviewer gives instructions aimed at aiding the participant to mentally reinstate both the physical and psychological context that existed at the time of encoding in line with the basic procedure currently taught to many police interviewers (see C. J. Dando, 2013, for verbatim MRC instructions). The instructions were delivered slowly and deliberately, and in between each instruction, the interviewer paused for five seconds to allow enough time for the participant to picture/image, and reinstate the context as instructed. The interviewer then gave the retrieval instructions.
  - In the Verbal-RC condition, the interviewer explained ‘In a moment, I am going to ask you to tell me what you remember about what happened, but before you begin think back to the video and say out loud the first word that comes to mind. Take your time. It doesn’t matter what the word is. Just say it.’ The interviewer repeats elements of this instruction a further 3 times, at which point the interviewer gives the retrieval instructions.
  - Irrespective of the condition, all participants were given unlimited time to explain what they remembered, during which time they were uninterrupted by the interviewer. Throughout, the interviewer displayed supportive and active listening behavior (nodding, smiling, looking up and down) while making brief bullet notes about the main topics remembered, and the order of those topics as they were verbalized by the interviewee (for use in the probing questioning phase).
iii. The probing questions phase immediately followed. All participants were again given the retrieval instructions prior to the commencement of this phase, during which the interviewer questioned participants, asking a tell, explain, or describe question about each of the topics recalled during the initial free recall phase, and in the same order. To do this, the interviewer used the notes made during that free recall phase. For example, if during the free recall a participant had mentioned a ‘woman’, during the probing questions phase the interviewer would say, ‘earlier you mentioned you saw a woman, I would like you to tell me everything you remember about that woman in as much detail as you can’.
iv. Thereafter, the interviewer completed the closure phase, during which the participant was thanked for his/her participation, debriefed, and offered an opportunity to ask questions and add any further information.

Before conducting interviews for this research, all interviewers underwent bespoke (designed for this research by the first author) training. Training adopted a collaborative pedagogical approach and comprised (i) a 3 h long classroom-based introduction to the protocols that were the subject of this research; (ii) a 4 h long practice session; (iii) reading of theoretical and applied training materials produced for this research; (iii) practice interviews (6 in total, 2 in each condition) which were digitally recorded to allow feedback and evaluation and (iv) instruction on reflective research practice and critical self-evaluation of performance. Once researchers had attended the training sessions and completed the required competencies (consistent and correct application of the protocols), they were able to commence research interviews. In total, training for this research took between 2 and 3 days to complete. Contact the first author for the interview protocol.

### 2.5. Post-Interview Questionnaire

The questionnaire comprised seven Likert-style questions concerning experiences and perceptions of ease/difficulty, memory performance, and confidence, etc. (ranging from 1 = very easy/very confident/very useful to 5 = very difficult/not at all confident/not at all useful). Post-interview feedback was collected via the Qualtrics research platform.

### 2.6. Procedure

Participants received a one-time link to watch the volume crime shop robbery on a laptop or desktop computer. Forty-eight hours later, participants were interviewed face-to-face, according to condition, using a verbatim protocol (randomly allocated). All participants were asked to complete an online post-interview questionnaire. Participants were all naive to the study design, aims, and hypotheses. Interviews were digitally audio and video recorded, transcribed verbatim, and coded for correct, errors of commission (information verbalized that is relevant to the witnessed episode but described with some error, e.g., describing a person’s brown jacket, but stating the jacket was black rather than brown), or confabulated (reporting information that was not present in the film) information recalled. Items recalled were only scored once (i.e., repetitions were not scored irrespective of interview phase). Twenty interviews from each condition were then randomly selected for coding by a further two independent coders, blind to the aims and hypotheses of the research but familiar with the method of scoring. Two-way mixed effects intraclass correlation coefficient (ICC) analysis testing for absolute agreement between coders for the overall amount of correct, errors of commission (here on referred to as errors, and confabulated recall were conducted. Mean estimations with 95% CI indicate good inter-rater reliability for correct information, ICC = 0.899 (95% CI, 0.593; 0.975), errors = 0.979 (95% CI 0.948; 0.992), and confabulations, ICC = 0.865 (95% CI 0.498; 0.964).

A selection of two interviews per condition for each of the 6 interviewers (36 in total) were coded for interviewer adherence to the condition-relevant verbal protocols. Each interview was coded by two independent coders, blind to the aims and hypotheses of the research. Coders scored each of the relevant behaviors as absent (scored 0), partially present (scored 1), and fully present (scored 2). Prior to coding, coders participated in a training session held by the first author during which the interview protocols and coding system were explained. Coders then practiced coding and discussed any disagreements/misunderstandings with the trainer to reach a consensus using the training interviews.

Irrespective of interview condition, all protocols included (i) engage and explain, (ii) retrieval instructions, (iii) free recall, (iv) questioning, and (v) closure, and each of the interviews included the correct retrieval condition instructions. Two-way mixed effects intraclass correlation coefficient (ICC) analysis testing for absolute agreement between coders indicated good/very good inter-rater reliability for all interviewer behaviors: engage and explain, ICC = 0.851 (95% CI, −0.415; 0.305), retrieval instructions ICC = 1.00 (95% CI, −0.388; 0.388), free recall, ICC = 0.920 (95% CI, −0.449; 0.362), questioning, ICC = 0.806 (95% CI, −0.500; 0.300), and closure, ICC = 0.892 (95% CI, −0.454; 0.345), Sketch-RC (where appropriate), ICC = 0.750 (95% CI, −0.944; 0.611), Verbal-RC (where appropriate), ICC = 0.899 (95% CI, 0.593; 0.975), and MRC (where appropriate), ICC = 0.778 (95% CI, −0.865; 0.579).

## 3. Results

### 3.1. Analysis Approach

A series of ANOVAs (applying Bonferroni’s correction by adjusting the alpha value to 0.017) were conducted to investigate condition (MRC, Sketch-RC, Verbal-RC) on global memory performance measures (correct, errors of commission and confabulations), and to support a more nuanced understanding of the locus of global results, performance across each of the two phases of the interviews was also analyzed (free recall and questioning). ANOVAs were followed by planned comparisons/contrasts that map onto the hypotheses formulated for this research (i) comparing the self-initiated retrieval cue condition (Sketch-RC + Verbal-RC) to the other-initiated retrieval cue condition (MRC) and (ii) comparing the self-initiated retrieval cue conditions, only (Sketch-RC to Verbal-RC). ANOVAs were conducted on the post-interview feedback data across conditions, followed by post hoc tests to explore significant effects. A series of ANOVAs revealed non-significant differences for global memory performance as a function of the interviewer, all *F*s, 2.406, all *p*s > 0.05; thus, the interviewer was not entered as a covariate.

### 3.2. Global Recall

There was a significant effect of retrieval condition for global correct recall, *F*(2, 146) = 47.109, *p* < 0.001, *ηp*^2^ = 0.39, errors, *F*(2, 146) = 6.808, *p* = 0.001, *ηp*^2^ = 0.09, and confabulations, *F*(2, 146) = 8.179, *p* < 0.001, *ηp*^2^ = 0.10 (see Table 1 for memory performance means, SDs and 95% CIs). There was a significant linear trend for correct and erroneous recall, indicating that these memory performance measures increased proportionally, *F*(1, 146) = 15.425, *p* < 0.001, and *F*(2, 146) = 12.205, *p* < 0.001, respectively. The linear trend for confabulations was non-significant, *F*(1, 146) = 0.416, *p* = 0.520.

Planned comparisons revealed that significantly less correct information was recalled by participants in the MRC (other-initiated retrieval cue condition) than by participants who self-initiated retrieval cues, *t* (146) = 7.789, *p* < 0.001, *d* = 2.68. Participants in the Sketch-RC self-condition recalled significantly more correct information than those in the Verbal-RC condition, *t* (146) = 5.748, *p* < 0.001, *d* = 2.73. Significantly fewer errors were also reported by participants who self-initiated retrieval cues than by participants in the MRC (other-initiated retrieval cue condition), *t* (146) = −3.625, *p* < 0.001, *d* = 1.27. There was a non-significant difference for the number of errors across the Sketch-RC and Verbal-RC conditions, *t* (146) = 0.707, *p* = 0.481, *d* = 0.14.

Participants who self-initiated retrieval cues also confabulated less than participants in the MRC (other-initiated retrieval cue condition), *t* (146) = −2.527, *p* = 0.013, *d* = 0.89. Participants in the Sketch-RC condition confabulated significantly less than those in the Verbal-RC condition, *t* (146) = −3.143, *p* = 0.002, *d* = 0.63 (see Table 1).

### 3.3. Retrieval Phase

#### Free Recall

There was a significant effect of retrieval condition for correct recall in the free recall phase, *F*(2, 146) = 44.022, *p* < 0.001, *ηp*^2^ = 0.38, errors, *F*(2, 146) = 13.287, *p* < 0.001, *ηp*^2^ = 0.15, and confabulations, *F*(2, 146) = 15.013, *p* < 0.001, *ηp*^2^ = 0.17 in the free recall phase (see Table 1 for memory performance means, SDs and 95% CIs). Planned comparisons revealed significantly less correct information was recalled by participants in the MRC condition than participants who self-initiated their retrieval cues (Sketch-RC + Verbal-RC), *t* (146) = 6.444, *p* < 0.001, *d* = 2.26. However, participants in the Sketch-RC condition recalled significantly more correct information than those in the Verbal-RC condition, *t* (146) = 6.748, *p* < 0.001, *d* = 1.35.

Significantly fewer errors were also reported by participants who self-initiated their retrieval cues (Sketch-RC + Verbal-RC) than participants in the MRC, *t* (146) = −4.879, *p* < 0.001, *d* = 1.71. There was a non-significant difference for the number of errors between the Sketch-RC and Verbal-RC self-initiated cues conditions, *t* (146) = −1.637, *p* = 0.104, *d* = 0.33. Participants who self-initiated their retrieval cues also confabulated less in the free recall than participants in the MRC, *t* (146) = −4.894, *p* < 0.001, *d* = 1.72. Participants in the Sketch-RC confabulated significantly less than those in the Verbal-RC condition, *t* (146) = −4.495, *p* = 0.016, *d* = 0.48 (see Table 1).

### 3.4. Questioning Phase

ANOVAs were non-significant across retrieval conditions in the questioning phase for correct recall, *F*(2, 146) = 1.701, *p* = 0.186, *ηp*^2^ = 0.02, errors, *F*(2, 146) = 1.782, *p* = 0.108, *ηp*^2^ = 0.03, and confabulations, *F*(2, 146) = 2.318, *p* = 0.102, *ηp*^2^ = 0.03 (see Table 1 for memory performance means, SDs and 95% CIs). Linear trends were also non-significant for all memory measures in this phase, all *F*s < 3.393, all *p*s > 0.067. Planned comparisons revealed a non-significant difference for correct recall in the question phase between the own-initiated retrieval cue conditions (Sketch-RC + Verbal-RC) and the self-initiated retrieval cue condition (MRC), *t*(146) = 1.560, *p* = 0.121, *d* = 0.55, and between the Sketch-RC and Verbal-RC self-initiated cue conditions, *t* (146) = −0.993, *p* = 0.322, *d* = 0.20.

There was a non-significant difference for errors in the question phase between the own-initiated retrieval cue conditions (Sketch-RC + Verbal-RC) and the self-initiated retrieval cue condition (MRC), *t* (146) = −0.746, *p* = 0.457, *d* = 0.26. However, participants in the Verbal-RC condition reported fewer errors than those in the Sketch-RC condition, *t* (146) = 1.993, *p* = 0.048, *d* = 0.40.

There was a non-significant difference for confabulations in the question phase between the own-initiated retrieval cue conditions (Sketch-RC + Verbal-RC) and the self-initiated retrieval cue condition (MRC), *t* (146) = 0.160, *p* = 0.873, *d* = 0.06. However, participants in the Verbal-RC condition confabulated more than those in the Sketch-RC condition, *t* (146) = −2.148, *p* = 0.033, *d* = 0.43.

### 3.5. Global Percentage Accuracy and Completeness

ANOVAs revealed significant effects of condition for accuracy, *F*(2, 146) = 23.957, *p* < 0.001, *ηp*^2^ = 0.25, and completeness, *F*(2, 146) = 48.552, *p* < 0.001, *ηp*^2^ = 0.40. Linear trends for both measures were significant, *F*(1, 146) = 20.270, *p* < 0.001, and *F*(1, 146) = 15.028, *p* < 0.001, respectively, indicating that these memory performance measures increased proportionally across conditions.

Participants in the own-initiated retrieval cue conditions (Sketch-RC + Verbal-RC) were significantly more accurate than those in the self-initiated retrieval cue condition (MRC), *t* (146) = 6.508, *p* < 0.001, *d* = 2.28. Participants in the Sketch-RC condition were significantly more accurate than those in the Verbal-RC condition, *t* (146) = 2.322, *p* = 0.022, *d* = 0.46. Participants in the own-initiated retrieval cue conditions (Sketch-RC + Verbal-RC) were significantly more complete than those in the other-initiated cue condition (MRC), *t* (146) = 7.835, *p* < 0.001, *d* = 2.74. Participants in the Sketch-RC condition were significantly more complete than those in the Verbal-RC condition, *t* (146) = 5.932, *p* < 0.01, *d* = 1.18.

### 3.6. Post-Interview Questionnaire

There were significant differences across conditions for how easy participants found it to remember the event, *F*(2, 144) = 7.346, *p* < 0.001, *ηp*^2^ = 0.09, and how easy participants found it to carry out the interviewer’s instructions for supporting episodic retrieval mode *F*(2, 146) = 14.352, *p* < 0.001, *ηp*^2^ = 0.16. Participants in the MRC condition reported finding it more difficult to remember (*M* = 3.12, SD = 1.02) than participants in the Sketch-RC (*M* = 2.50, SD = 0.91) and Verbal-RC conditions (*M* = 2.48, SD = 0.69), *p* < 0.001, with a non-significant difference between the latter two conditions, *p* = 0.994. Participants in the MRC condition also reported finding it more difficult to carry out the retrieval instructions (*M* = 3.52, SD = 1.13) than participants in the Sketch-RC (*M* = 2.47, SD = 1.10) and Verbal-RC conditions (*M* = 2.50, SD = 1.11), *p* < 0.001. Again, there was a non-significant difference between the latter two conditions, *p* = 0.990.

There were non-significant differences across conditions for the remaining five post-interview questions. All participants ‘definitely’ understood the instructions given by the interviewer, *F*(2, 146) = 0.083, *p* = 0.920, *ηp*^2^ = 0.01 (*M*_Verbal-RC_ = 1.58, SD = 0.64; *M*_Sketch-RC_ = 1.61, SD = 0.64; *M*_MRC_ = 1.65, SD = 0.67). Participants all found the interviewer ‘very’ friendly, *F*(2, 146) = 0.020, *p* = 0.980, *ηp*^2^ = 0.89 (*M*_Verbal-RC_ = 1.44, SD = 0.50; *M*_Sketch-RC_ = 1.43, SD = 0.50; *M*_MRC_ = 1.42, SD = 0.49). Participants were all ‘somewhat’ confident that they remembered a lot, *F*(2, 146) = 0.008, *p* = 0.992, *ηp*^2^ = 0.91 (*M*_Sketch-RC_ = 3.55, SD = 1.14; *M*_MRC_ = 3.56, SD = 1.13, *M*_Verbal-RC_ = 3.58, SD = 1.14), and ‘somewhat’ confident that what they remembered was correct, *F*(2, 145) = 0.438, *p* = 0.646, *ηp*^2^ = 0.16 (*M*_Verbal-RC_ = 3.58, SD = 1.14; *M*_Sketch-RC_ = 3.55, SD = 1.14; *M*_MRC_ = 3.56, SD = 1.13). Participants in all conditions reported finding the condition relevant technique for supporting episodic retrieval mode, similarly ‘useful’ for helping them to remember (*M*_Sketch-RC_ = 1.92, SD = 0.95, *M*_Verbal-RC_ = 2.04, SD = 0.86; *M*_MRC_ = 2.00, SD = 0.83), *F*(2, 146) = 0.244, *p* = 0.784, *ηp*^2^ = 0.03.

## 4. Discussion

Criminal justice systems worldwide (CJS) rely on information from witnesses who typically explain what they remember during an interview. Retrieving information from episodic memory is cognitively demanding. Thus, many witness interview protocols include mnemonic techniques for facilitating contextual recall of past events, which is essential for obtaining detailed and accurate witness accounts (e.g., Lepage et al., 2000; Rugg & Vilberg, 2013; Danker & Anderson, 2010). Thus, developing a toolkit of effective witness-appropriate techniques for supporting *all* witnesses to access consciously remembered personally experienced events may enhance investigative outcomes, simultaneously promoting inclusivity.

Here, we compared self-generated cues (verbal and sketching) to the prevailing other-generated cues technique, namely the Mental-Reinstatement-of-Context (MRC) method. Here, we expected both self-generated cues to be more effective than MRC for cueing the retrieval of the to-be-remembered event (H^1^). Self-generated cues, by their very nature, are individually salient. Accordingly, it was sensible to expect they would be more effective, whereas the MRC technique provides blanket retrieval cues, which may be less or not at all salient and thus possibly less effective for facilitating contextual recall. Further, we expected the Sketch-RC technique would improve memory performance versus Verbal-RC (H^2^) simply because drawing engages both visual-spatial and verbal memory systems, and as such, it is reasonable to argue that sketching might trigger richer contextual reinstatement.

Overall, our results support both hypotheses, but with some limitations. Global memory performance reveals participants in the self-generated cue conditions recalled an average of 26% (Sketch-RC) and 11% (Verbal-RC) more correct information and were more complete and more accurate than those in the MRC condition. This improved recall was not accompanied by a mean increase in errors or confabulations. Indeed, combined mean errors (errors + confabulations) were an average of 22% (Verbal-RC) and 34% (Sketch-RC) lower in the self-generated cue conditions than in the MRC. That said, as is typical in mock witness laboratory research, errors were few per se, and while these differences were statistically significant, they equate to around just two information items (mean range 1.67 to 2.29). Confabulations were also few (mean range from 0.80 to 1.5) and were non-significant across conditions. Reducing all types of erroneous recall is nonetheless important since even small decreases in inaccurate recall can meaningfully improve the overall reliability of witness accounts and strengthen the evidential value of the information they provide. That improved recall was not accompanied by increased confabulations adds further to the strength of both self-initiated cue techniques.

Globally, the Sketch-RC technique was most effective. Participants recalled more correct information and confabulated less than those in the Verbal-RC and thus were significantly more accurate. Sketch-RC participants were 14% more complete and 28% more complete than those in the Verbal-RC and MRC conditions, respectively. This pattern of results sits well with predictions from the neurocognitive literature and the theoretical value of self-initiated cues for facilitating episodic retrieval mode through reactivation of encoding-relevant neural traces (e.g., Choucry et al., 2024; Richter et al., 2016; Rugg & Vilberg, 2013; Danker & Anderson, 2010). Participants reported finding the MRC instructions the most challenging interview, and also found remembering more difficult, which suggests the cognitive demands of the MRC technique potentially diverts finite cognitive resources away from entering and sustaining episodic retrieval mode (C. J. Dando et al., 2011, 2015; Miller & Unsworth, 2018; Unsworth & Engle, 2007). Our findings are also consistent with prior laboratory research demonstrating that self-generated retrieval support, including sketch-based techniques, can yield more detailed and accurate recall in some instances than interviewer-provided cues (e.g., C. Dando et al., 2009; C. J. Dando et al., 2020, 2023b; M. L. Mattison et al., 2015; M. Mattison et al., 2018; Matsuo & Miura, 2017; Milne & Bull, 2002; Wheeler-Mundy et al., 2025).

Witness interviews, however, are more than the sum of their parts (Fisher & Geiselman, 1992; Griffiths & Milne, 2010; Memon et al., 2010), and so analysis of each of the individual retrieval phases offers further insights into the locus of the global effects. A similar pattern emerged for the initial free recall and the questioning phases, as was evident in the global performance results. Both self-generated cue conditions consistently outperformed MRC in the amount of correct information recalled, without a corresponding increase in errors or confabulations. Although effect magnitudes varied slightly across phases, the overall advantage for self-generated cueing appears robust.

Sketch-RC outperformed Verbal-RC in the free recall, leveraging over 30% more correct information than both MRC and Verbal-RC in this phase. Errors were similar and consistently low across both self-initiated cue conditions in the free recall and questioning phases. However, Sketch-RC resulted in fewer confabulations in the free recall phase than participants in the Verbal-RC, but vice versa in the questioning phase, albeit confabulations were less than one in both phases, so one might argue are negligible. Nonetheless, these subtle performance patterns shed light on how different forms of self-generated cueing shape episodic retrieval across interview phases, towards the development of optimally supportive interviewing practices.

### Limitations

Mock eyewitness research of this nature is necessarily limited by a lack of ecological validity, because staged or video-based events cannot reproduce the emotional intensity, stress, and sensory complexity of real crimes. Participants typically view events under controlled, interruption-free conditions of intentional coding and are often aware that they will later be asked to recall what they observed, as was the case here. Furthermore, mock interview contexts rarely capture the interpersonal and procedural complexities of witness interviews, such as the social pressures that can shape witness responding. However, laboratory research is important in terms of tight manipulation of task demands, retrieval support, and interview/interviewer variables, thereby generating foundational knowledge for developments in applied investigative contexts.

We sought to counter some limitations by using several interviewers. Albeit for pragmatic reasons, all interviewers were female, thus introducing naturally occurring interviewer communication style variability. Further, we employed the current best practice investigative interview protocol for use with witnesses of volume-type crime. Our video depicted a multi-actor incident, and we reduced anticipatory encoding by masking study aims. Finally, we recruited a community-based sample to improve generalizability. No participant had been interviewed by police as a witness, so they did not know what to expect, and had never previously met any of the interviewers, which arguably heightens cognitive and situational demands, particularly since mock-witnesses generally want to perform ‘well’. Finally, our sample size was slightly fewer (149) than a priori power analysis indicated was necessary (155) for a medium effect size.

## 5. Conclusions

Our results again highlight the importance of transferring greater control of the retrieval process to witnesses while simultaneously providing appropriately targeted retrieval support. Further work is needed to examine efficacy across diverse witness groups and in more ecologically valid encoding contexts. Larger sample sizes and replication research that considers the type of recall (actions, objects, persons, etc.) and includes analysis of the cue words produced and the sketches drawn, which would offer further insights. However, both self-initiated cue conditions naturally allow witness-compatible adjustments, aligning well with the task support hypothesis, and offer interviewers criminal-justice-appropriate, straightforward techniques for facilitating recall. Allowing witnesses to dictate the pace of their remembering can reduce both situational and cognitive demands, thereby supporting more effective episodic retrieval. Consistent with prior theoretical and empirical work, self-generated cues were more effective than other-initiated cues and were well received; both Sketch-RC and Verbal-RC conferred clear advantages, although here Sketch-RC was the most efficient technique overall. For witnesses unable or unwilling to sketch, Verbal-RC may be a viable alternative.

## Figures and Tables

**Table 1:** Means (SD) and [95% CI] for global and phase memory performance (correct, errors of commission, confabulations) and percentage accuracy and completeness across conditions (Sketch-RC; Verbal-RC; MRC).

	Sketch-RC	Verbal-RC	MRC
	Mean (SD) [95% CI]		
Global Memory			
Correct	25.40 (2.79) [24.60, 26.20]	22.40 (2.72) [21.60, 23.10]	20.20 (2.53) [19.50, 21.00]
Errors	1.71 (0.93) [1.45, 1.97]	1.56 (1.22) [1.21, 1.91]	2.29 (0.94) [2.02, 2.57]
Confabulations	0.80 (0.87) [0.56, 1.05]	1.38 (1.02) [ 1.09, 1.67]	1.50 (0.85) [1.25, 1.75]
Free Recall Phase			
Correct	15.75 (3.11) [14.87, 16.62]	12.18 (2.43) [11.49, 12.87]	10.98 (2.28) [10.32, 11.64]
Errors	0.33 (0.48) [0.20, 0.47]	0.54 (0.71) [0.34, 0.74]	0.98 (0.70) [0.78, 1.18]
Confabulations	0.16 (0.37) [0.05, 0.26]	0.40 (0.54) [0.25, 0.55]	0.71 (0.58) [0.54, 0.88]
Probing Question			
Correct	9.69 (2.25) [9.05, 10.32]	10.18 (2.75) [9.40, 10.96]	9.25 (2.47) [8.53, 9.97]
Errors	1.37 (0.82) [1.14, 1.60]	1.02 (1.00) [0.74, 1.30]	1.31 (0.83) [1.07, 1.55]
Confabulations	0.65 (0.82) [0.42, 0.88]	0.98 (0.87) [0.73, 1.23]	0.79 (0.62) [0.61, 0.97]
Percentage Accuracy	91.16 (4.39) [89.92, 92.40]	88.79 (5.56) [87.21, 90.37]	84.12 (5.39) [82.56, 85.68]
Completeness	41.08 (4.52) [39.81, 42.35]	36.00 (4.29) [34.78, 37.22]	32.63 (4.08) [31.44, 33.81]

## Data Availability

Raw data files are available at OSF (https://osf.io/25m7v/overview?view_only=f7bf236d4c4b4dceb382be18418f2fa1, accessed on 1 October 2025).

## Note

1 From here on, we use the terms witness and eyewitness to refer to witnesses, victims, and survivors of crime.

## Abbreviations

The following abbreviations are used in this manuscript:

ANOVA	Analysis of Variance
